# Comparison of phenotypic selection of inbred lines, genomic selection of inbred lines, and evolutionary populations for field pea breeding in three Mediterranean regions

**DOI:** 10.3389/fpls.2025.1565087

**Published:** 2025-06-17

**Authors:** Paolo Annicchiarico, Meriem Laouar, Imane Thami-Alami, Margherita Crosta, Nelson Nazzicari, Luciano Pecetti, Luigi Russi

**Affiliations:** ^1^ Research Centre for Animal Production and Aquaculture, Council for Agricultural Research and Economics (CREA), Lodi, Italy; ^2^ Laboratory of Integrative Improvement of Plant Production, Ecole Nationale Supérieure Agronomique (ENSA), Algiers, Algeria; ^3^ Centre Régional de Rabat, Institut National de la Recherche Agronomique (INRA), Rabat, Morocco; ^4^ Department of Agricultural, Food and Environmental Science, University of Perugia, Perugia, Italy

**Keywords:** bulk breeding, drought tolerance, evolutionary breeding, heterogeneous material, intercropping, intra-species diversity, *Pisum sativum*, yield stability

## Abstract

Pea breeding may rely on phenotypic selection (PS) of single-seed descent (SSD) or bulk-derived lines, line genomic selection (GS), and selection of evolutionary populations (EPs). This study aimed to compare all these approaches in region-specific grain yield selection for Central Italy, coastal Algeria, and inland Morocco, using material from three connected crosses of elite, geographically diverse parent cultivars. Bulk breeding and initial EP development were based on three-year mass selection for plant yield under managed severe drought for Algeria and Morocco, and four-year field-based natural and mass selection under autumn sowing in Northern Italy for Central Italy. One EP per region was eventually developed by additional region-specific three-year field-based natural selection on pooled three-cross material. Region-specific line selections were performed on each cross using three experiments per region for PS, and GS models developed in previous studies. An additional GS was performed for a putative Stressful Italy region by combining predictions for Italy and Morocco. In a comparison of top-performing bulk-derived lines vs. SSD-derived lines, the former out-yielded the latter by at least 10% in four environments featuring the same predominant stress (drought or low winter temperatures) that had acted on bulked progenies. All selections were evaluated in one location per target region for two cropping years, in a managed drought environment, and in intercropping with barley in Algeria and Morocco. Results indicated that: (a) EPs did not differ from GS- or PS-derived lines for mean yield in their target regions while showing greater yield stability, better response to a climatically unfavorable year, and broader adaptability to intercropping or other non-target environments; (b) EPs were out-yielded by the top-yielding line in each target region; (c) GS- and PS-derived lines did not differ in mean yield or yield stability, but a superiority of GS over PS emerged for Algeria and tended to emerge for Morocco when comparing the top-yielding lines; (d) GS-derived lines for Stressful Italy displayed comparable mean yield and higher yield stability than other region-specific GS-derived lines. Our results suggested different opportunities for adopting and integrating biotechnology- and agroecology-based approaches depending on the characteristics and objectives of the breeding program.

## Introduction

The Mediterranean region is particularly exposed to the impacts of global warming, which is causing more severe and prolonged droughts and heat waves along with wider year-to-year variation in rainfall and temperature patterns (Urdiales-Flores et al., 2023). In this region, food and feed insecurity are driven by population growth, reduced availability of irrigation water, land degradation caused by overgrazing and continuous cereal cropping, and inefficient and environmentally damaging N fluxes (Kepner et al., 2006; Turral et al., 2011; Voltz et al., 2018; Antonelli et al., 2022), in a context of strong dependency on imported high-protein feedstuff (FAO, 2010; Pilorgé and Muel, 2016). Greater legume cultivation could be pivotal for Mediterranean countries to increase the sustainability of their agri-food systems (Cellier et al., 2015; Foyer et al., 2016). While the agro-economic advantage of growing legumes is often underestimated (Preissel et al., 2015; Yigezu et al., 2019), the profitability gap between these crops and major cereals remains the main reason for their modest cultivation (Magrini et al., 2016). Increased plant breeding efforts have been advocated as the main avenue to reduce this gap (Magrini et al., 2016; Rubiales et al., 2021).

Field pea (*Pisum sativum* L.) is a cool-season grain legume with high potential interest for Mediterranean crop-animal systems, owing to its high yielding ability and its flexibility of utilization (as grain, hay, or silage). This crop featured greater production of grain and feed energy per unit area than other cool-season grain legumes in Southern Europe (Annicchiarico, 2008) and Western Europe (Carrouée et al., 2003), and tended to out-perform other annual legumes for dry biomass yield in three Mediterranean regions (Algeria, Morocco, and Sardinia) (Annicchiarico et al., 2017c). In addition, pea is highly suitable for producing protein concentrates or isolates used in the industry of vegetarian or vegan foods, owing to its nutritive value, lack of gluten, low allergenicity, and absence of GM cultivars (Lam et al., 2018). Field pea has displayed moderately high rates of genetic yield gain (Warkentin et al., 2015), also due to increased lodging tolerance enabled by the adoption of a semi-dwarf, semi-leafless plant type. However, pea breeding for drought-prone Mediterranean regions has largely been neglected (Bagheri et al., 2023), as confirmed by the higher mean yield of a large set of international landraces compared with recent European varieties in a stressful Italian environment (Annicchiarico et al., 2017b).

The opportunity for extensive, low-cost genotyping has opened new scenarios for pea selection of genetically complex traits, encouraging the definition of genomic selection (GS) models (Lorenz et al., 2011) to shorten the selection cycles and reduce the extent of field-based selection (Li et al., 2022). A pioneer study based on SNP array data revealed a good ability to predict seed yield components in a pea germplasm collection (Burstin et al., 2015). In later studies mostly based on genotyping-by-sequencing (GBS)-generated SNP markers (Elshire et al., 2011), GS exhibited moderately high predictive ability for key traits such as grain yield and protein content (Annicchiarico et al., 2017a; Annicchiarico et al., 2019b; Annicchiarico et al., 2020; Al Bari et al., 2021; Crosta et al., 2022; Crosta et al., 2023; Saludares et al., 2024; Crosta et al., 2025). Other promising target traits for pea GS include adaptation to intercropping with cereals (Annicchiarico et al., 2021), which can promote an agroecology-based intensification of low-input systems (Bedoussac et al., 2014; Vonzun et al., 2024), and the resistance to biotic stresses (Carpenter et al., 2018; Osuna-Caballero et al., 2024). Grain yield predictions (especially if applied to the same genetic base used for model construction) indicated an advantage of GS over phenotypic selection (PS) in terms of predicted genetic gain per unit time (Annicchiarico et al., 2019b). However, no comparison of GS vs. PS based on actual yield gains is available, except for a proof-of-concept study showing comparable gains per selection cycle under managed severe drought (Annicchiarico et al., 2020).

**Figure 1:**
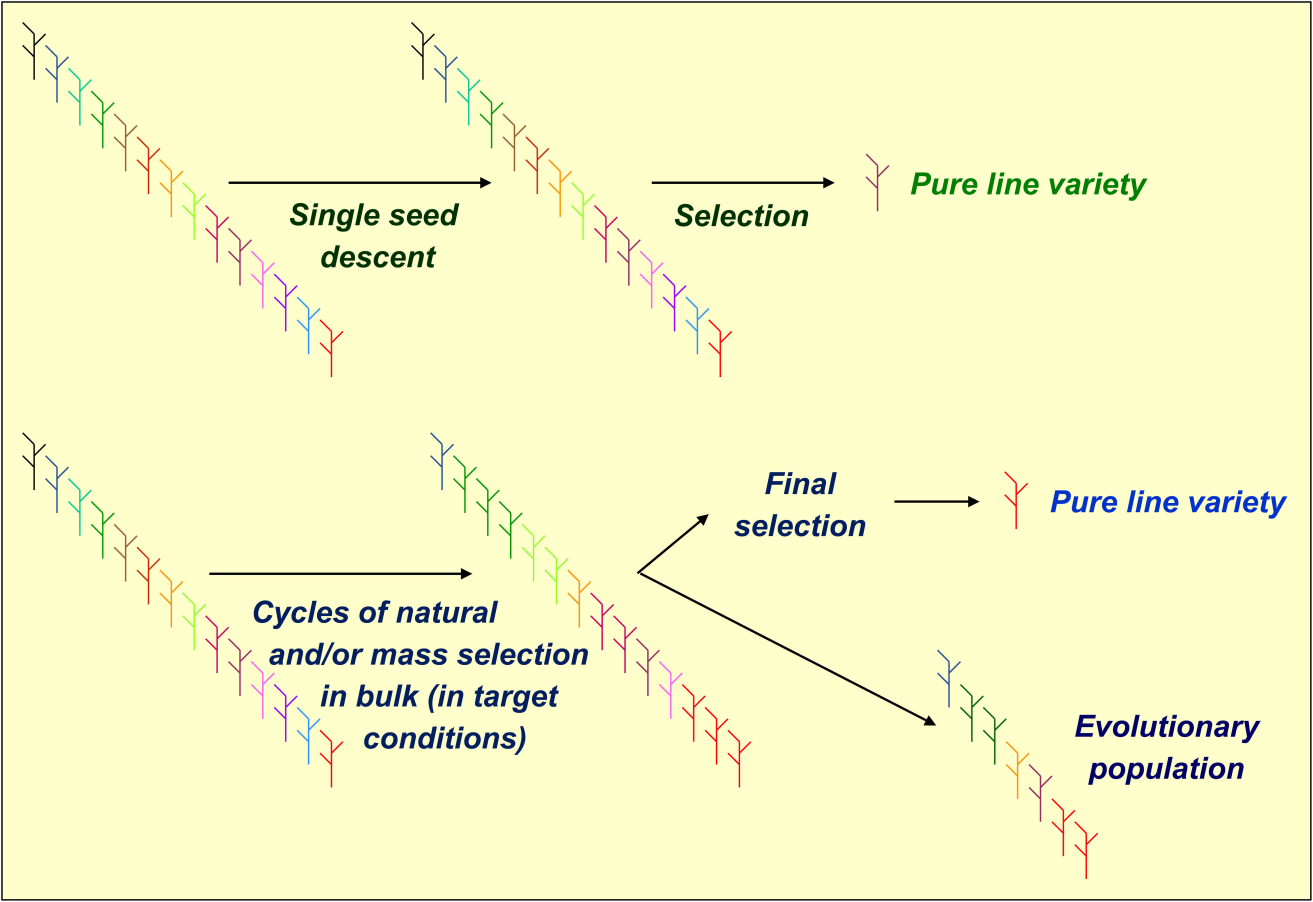
Graphical representation of pea selection schemes for single-seed descent or bulk-derived inbred lines (using phenotypic or genomic selection) and evolutionary populations.

Phenotypic or genomic selections are ordinarily applied to inbred lines produced by single-seed descent (SSD). However, they may also be applied to lines issued by bulk breeding, which exploits natural or mass selection of early segregating material grown in well-defined, target conditions (**Figure 1**). Compared to SSD, bulk breeding is less expensive, allows for producing large numbers of inbred lines, and may increase the frequency of genotypes adapted to specific environments. On the other hand, it delays the production of inbred lines by preventing the performance of off-season generations (Simmonds, 1979; Ranalli and Cubero, 1997). Earlier comparisons of SSD-based vs. bulk-based breeding for cool-season grain legumes were few and provided inconsistent results. SSD showed an advantage for pea in India (Kumar et al., 1992) and for lentil in North-Western USA (Haddad and Muehlbauer, 1981), whereas bulk-based breeding showed an advantage for chickpea in India (Meena and Kumar, 2012) and for pea in Northern and Central Italy (Annicchiarico et al., 2023).

Modern agriculture caused a substantial genetic erosion of cultivated inbred crops by replacing landrace populations with a small number of genetically homogeneous cultivars grown across vast areas (Van de Wouw et al., 2009). Reintroducing genetically heterogeneous cultivars may counterbalance this trend and stabilize the crop yields across environments characterized by increasing climate unpredictability (Döring et al., 2011; Ceccarelli and Grando, 2020). Harlan and Martini (1929) and Suneson (1956) proposed to develop evolutionary population (EP) material by pooling equal amounts of progeny seed issued from various crosses and letting the population evolve across several generations under natural selection in specific growing conditions. The selection of EPs, which generally involves definitely lower costs than that of inbred lines, may also combine mass and natural selection (Murphy et al., 2005; Phillips and Wolfe, 2005). Bulk breeding may represent a step for selecting EPs originated from one or a few crosses (**Figure 1**). While even biparental EPs may be valuable (Merrick et al., 2020), the EPs originated from many parents may fail to display sufficient yielding ability compared with locally best-adapted inbred lines because of the genetic load caused by poorly adapted parent material (Döring et al., 2015; Brumlop et al., 2017). Studies on small-grain cereals usually indicated greater yield stability along with similar or somewhat lower mean yield of EPs relative to top-performing parent lines or commercial cultivars (e.g., Döring et al., 2015; Bocci et al., 2020; Goldringer et al., 2020; Merrick et al., 2020; Baresel et al., 2022). This result was confirmed for wheat intercropped with pea (Timaeus et al., 2022). In a previous study on pea, EPs and top-yielding bulk-derived lines selected from the same genetic base displayed comparable yielding ability and yield stability across Northern and Central Italy (Annicchiarico et al., 2023). The marketing of EPs is allowed for organic farming in the EU since 2022, while being limited to informal seed systems elsewhere (Joshi et al., 2024).

Our study aimed at comparing various selection strategies for pea grain yield, encompassing PS or GS of inbred lines issued from SSD or bulk breeding, and the selection of EPs. All materials derived from the same genetic base of elite parent cultivars. The comparison was carried out for each of three Mediterranean regions: Central Italy, coastal Algeria (referred to as Algeria hereafter), and inland Morocco (referred to as Morocco hereafter). Selections were region-specific, owing to large genotype × environment interaction (GEI) that emerged even across North African regions (Annicchiarico et al., 2020). All the selections were evaluated in the three target regions, as well as under managed drought in Northern Italy. Adaptation to intercropping with barley was also assessed limitedly to selections for North African countries, to highlight the possible advantage of genetic heterogeneity for a cropping environment that was not targeted by selection.

## Materials and methods

### Generation of inbred lines and selection of evolutionary populations

The genetic base of all selections derived from paired crosses between three semi-dwarf, semi-leafless cultivars: Attika (a European cultivar described as a spring-type), Isard (a French winter-type cultivar), and Kaspa (an Australian cultivar of Mediterranean type). These cultivars revealed high and stable grain yield and modest difference in crop maturity across environments of Northern and Southern Italy (Annicchiarico, 2005; Annicchiarico and Iannucci, 2008).

**Figure 2:**
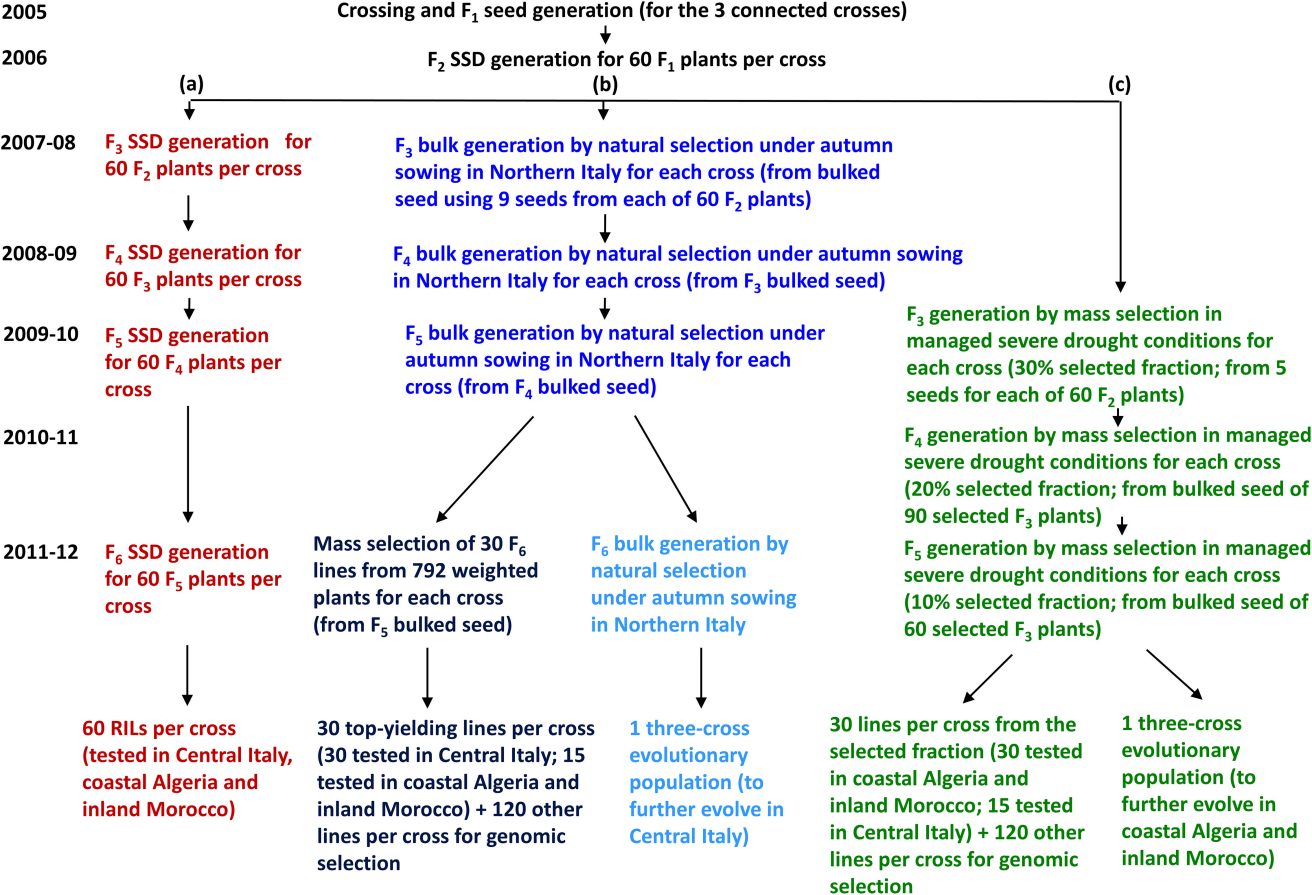
Generation of material from three pea connected crosses for selection in three target regions: **(a)** single-seed descent (SSD)-derived inbred lines (in red), **(b)** bulk-derived inbred lines and a three-cross evolutionary population (EP) for Central Italy (in blue), and **(c)** bulk-derived inbred lines and a three-cross EP for Algeria and Morocco (in green).

From each of the three crosses, we generated a recombinant inbred line (RIL) population of 60 F_6_ lines via SSD [path (a) in **Figure 2**]. Bulk-derived inbred lines and EPs originated jointly by initial cycles of natural and/or mass selection (**Figure 1**) that were separate for each cross and were performed under different growing conditions for material targeted to Italy or to North African countries. For both types of material, the first cycle of bulk selection originated from bulked seed supplied in equal amount by 60 F_2_ plants per cross. After several cycles of bulk selection, we developed one EP for Italy and one for North African countries by pooling equal amounts of seed from the EPs originated from the three crosses that evolved in the relevant conditions. Then, the pooled three-cross EP for North African countries was split, to undergo separate selection in Algeria and Morocco.

Bulk breeding for Italy targeted both Central and Northern Italy, considered as one target region according to earlier studies showing that GEI for pea grain yield in Italy is more affected by year-to-year climate variation than by the geographical distance of the growing sites (Annicchiarico and Iannucci, 2008; Pecetti et al., 2019). It should be noted, however, that the climate in Northern Italy tends to be wetter and colder than in Central Italy. Bulk breeding procedures are described in detail in Annicchiarico et al. (2023). In brief, for each cross we performed four cycles of natural selection under autumn-sown field conditions in Lodi, Northern Italy (45°19′ N, 9°30′ E), favoring a balanced intra-specific plant competition by adopting a consistent sowing depth and plant spacing (by a pneumatic seed drill), chemical weed control, and border plants. A mass selection based on individual plant seed yield was performed in the fourth growing cycle on a random sample of 792 plants per cross. We selected the 30 top-yielding F_6_ plants per cross for field testing aimed at PS and GS model construction, and 120 lower-ranking plants per cross for GS application [path (b) in **Figure 2**]. The EP included the seed of all bulk-selected and non-selected lines in proportion to their contribution to the harvested seed.

Bulk breeding for Algeria and Morocco was performed in Lodi in a large sheltered artificial environment equipped with micro-sprinklers. Mass selection under autumn sowing was conducted for three selection cycles under managed severe drought as reported in detail by Annicchiarico et al. (2023). In brief, plant material from each cross was grown in 30 adjacent grids of 10 plants each, imposing increasing drought stress and within-grid selection intensity for plant seed yield across selection cycles. The seed of mass-selected plants after the first or second cycle were pooled for the following cycle. In the third cycle, the top-yielding plant in each grid (i.e., the 10% selected fraction) was selected to produce, overall, 30 F_5_ inbred lines per cross for field testing aimed to PS and GS model construction, while 120 lower-ranking lines per cross were retained for GS application [path (c) in **Figure 2**]. The EP selected for each cross after the third selection cycle included the 150 mass-selected lines of the cross in a proportion that reflected the yield of each plant. The current bulk selection differed from that for Italy for the significantly lower quantity of available water (averaging 130 mm vs. 405 mm over the December-May period) and the average temperature of the sheltered environment which was approximately 2°C higher.

**Figure 3:**
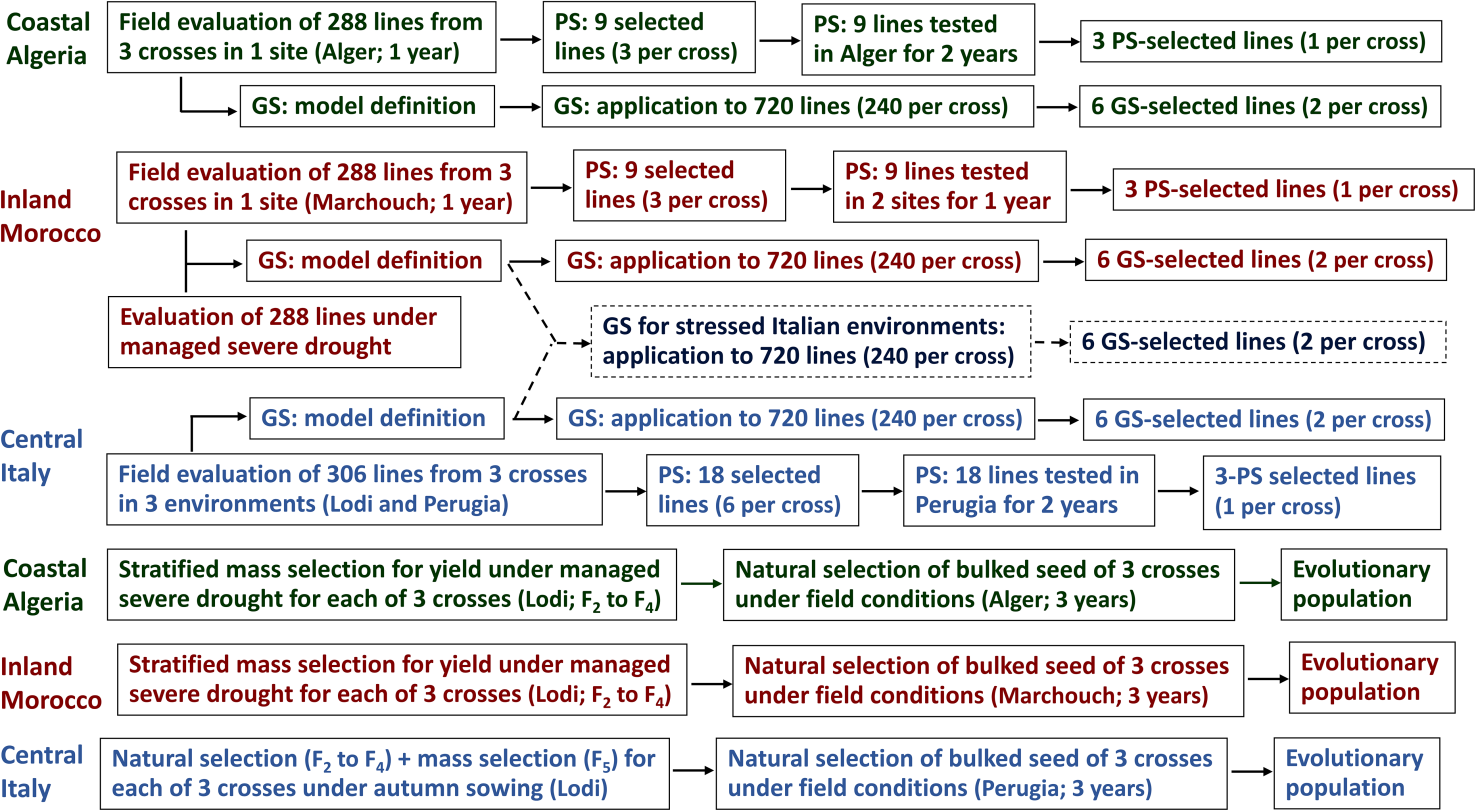
Generation of genomically-selected and phenotypically-selected inbred lines and evolutionary populations targeted to Algerian, Moroccan and Italian regions.

The EP for Italy, obtained by pooling the EPs from the three crosses generated by field-based natural and mass selection in Northern Italy, was subjected to field-based natural selection under autumn sowing in Perugia (Central Italy; 43°06′N, 12°23′E) for three cropping years (2014-15, 2015-16, and 2018-19), adopting an organic crop management. The EP for North African countries, obtained by pooling the EPs of the three crosses issued by mass selection under managed severe drought, was subjected to a three-year field-based natural selection under autumn sowing separately in Algiers (coastal Algeria, 36°45′N, 3°3′E; seasons 2015-16, 2017-18, and 2018-19) and Marchouch (inland Morocco, 33°33′N, 6°41′W; seasons 2016-17, 2017-18, and 2018-19) (**Figure 3**). All these EPs were mechanically sown and harvested in bulk.

### Phenotypic selection of inbred lines and comparison of SSD-derived vs. bulk-derived lines

A set of experiments assessed the grain yield of 315 lines (105 from each cross) with the following aims: (a) comparing SSD-derived vs. bulk-derived inbred lines; (b) providing the first step of region-specific PS; and (c) generating phenotyping data for the development of region-specific GS models. While the first aim fulfilled one of the objectives of this study, the second and third aims contributed to the comparison of PS vs. GS that is described later on.

The field experiments in Italy included 60 SSD-derived lines per cross, 30 top-yielding bulk-derived lines per cross issued from natural and mass selection in autumn-sown environments of Northern Italy, and 15 top-yielding bulk-derived lines issued from mass selection under managed drought (out of 30). These lines were evaluated for grain yield under autumn sowing in Lodi for two cropping years (2013–14 and 2014-15) and in Perugia for one year (2013-14). The experiments were conducted in randomized complete block (RCB) designs with three replications as described in Annicchiarico et al. (2019b) (where all lines are generically referenced as RILs). The trials in 2013–14 adopted an organic crop management. We selected phenotypically six lines per cross based on mean yield across environments, and built a GS model for Italy, using data of 306 lines (as nine lines displayed poor quality of phenotypic and/or genotypic data). The 18 top-yielding lines obtained from this first PS stage were subjected to additional yield evaluation for PS in Perugia during the seasons 2014–15 and 2015–16 in organically managed trials with three replications described as Experiments 5 and 7 in Annicchiarico et al. (2023). **Table 1** reports rainfall and absolute minimum temperature of these trials (referenced as environment 2 and 3 for PS). The ultimate phenotypically-selected lines for Central Italy included the top-performing line of each cross based on mean yield across the three test years of Perugia (**Figure 3**).

**Table 1:** Water available for the crop (WA, mm) from December to May and absolute minimum temperature across the crop cycle (AMT, °C) for environments used in the definition of genomic selection (GS) models, phenotypic selection (PS), development of region-specific evolutionary populations, and evaluation of genomically- and phenotypically-selected lines, evolutionary populations and parent cultivars.

	Algiers (Algeria)^a^		Marchouch (Morocco)^a^		Perugia (Italy)^b^		Lodi (Italy)		Managed drought (Lodi)	
Evaluation target	WA	AMT	WA	AMT	WA	AMT	WA	AMT	WA	AMT
GS definition + PS environment 1^c^	348	0.6	168	1.9	465	−3.6	467	−8.7	120	−
PS environment 2	500	−0.3	410	0.1	340	−2.9	−	−	−	−
PS environment 3	271	0.0	197	−2.5	415	−5.4	−	−	−	−
Regional EP development (average)	373	0.1	204	−0.2	375	−4.4	−	−	−	−
Evaluation season 2021-22	352	−0.8	185	−1.8	299	−3.7	−	−	230	−
Evaluation season 2022-23	296	0.7	230	−0.8	405	−4.1	−	−	−	−

a Initial bulk breeding and development of the evolutionary populations for these locations took place in Lodi under managed severe drought over three years, with average WA from December to May of 130 mm.

b Initial bulk breeding and development of the evolutionary population for this location took place in Lodi under autumn-sown field conditions over four years, with average WA from December to May of 405 mm and average AMT of −12.0°C.

c These environments were also used for comparison of bulk-derived vs. single-seed descent-derived inbred lines. Applied GS models were specific for: (i) coastal Algeria (based on data from Algiers), (ii) inland Morocco (based on data from Marchouch and managed drought), (iii) Italy (based on data from two environments in Lodi, Northern Italy, and one environment in Perugia, Central Italy), and (iv) Stressful Italy, based on the combination of the models (ii) and (iii).

The experiments performed in Algeria and Morocco included 60 SSD-derived lines per cross, 30 top-yielding bulk-derived lines per cross issued from mass selection under managed drought, and 15 top-yielding bulk-derived lines per cross issued from natural and mass selection under field conditions in Northern Italy (out of 30). These lines were evaluated for grain yield in Algiers and in Marchouch during the season 2015–16 in autumn-sown RCB experiments with three replications as described in Annicchiarico et al. (2020). Due to poor quality of phenotypic or genotypic data of some lines, we used 288 lines for GS model construction and for region-specific PS of three lines per cross for further region-specific PS experiments. The selected lines were evaluated: (a) in Algiers in the seasons 2017–18 and 2018–19 in RCB experiments with four replications; (b) in Marchouch and in Jemaate Shim (32°21′ N, 8°51′ W) in the season 2017–18 in lattice experiments with three replications. **Table 1** reports rainfall and absolute minimum temperature also for these trials (PS environments 2 and 3 in the table). The region-specific PS retained the top-yielding line of each cross based on mean yield across the three test environments per country (**Figure 3**).

In addition, we performed a managed severe drought experiment in Lodi in the environment previously used for bulk selection. This experiment, which is described in Annicchiarico et al. (2017a), provided an additional environment for comparing SSD- vs. bulk-derived lines, and contributed to define GS models.

### Genomic selection of inbred lines

The lines used for definition or application of GS models for grain yield were genotyped by GBS according to Elshire et al.’s (2011) method with modifications, as described in Annicchiarico et al. (2019b) along with details on the adopted SNP calling and data filtering procedures. In the joint SNP calling for the training and selection data sets, we called the SNP markers according to the reference genome (Kreplak et al., 2019). We developed region-specific GS models for inbred lines of each of the three crosses as described in earlier reports for Italy (Annicchiarico et al., 2019b) and the two North-African regions (Annicchiarico et al., 2020). GS model selection envisaged two possible statistical models, i.e., Ridge Regression BLUP and Bayesian Lasso, and different possible rates of missing data per marker. We assessed the model predictive ability (as correlation between predicted and observed values) for each of the three crosses by 10-fold cross validations averaged across 10 analyses separately for each model and configuration of missing data, using the R package GROAN (Nazzicari and Biscarini, 2017). The GS model ultimately selected for each region maximized the model predictive ability averaged across values for the three crosses. The selected GS model for Northern and Central Italy (considered as one region), which was based on phenotyping data of 306 lines averaged across two environments of Lodi and one of Perugia, was Bayesian Lasso including up to 4,966 markers per cross (the marker number depending on the selected marker missing rate and the within-cross marker polymorphism) (Annicchiarico et al., 2019b). This model showed an average intra-environment, intra-cross predictive ability of 0.48. The definition of GS models for Algeria and Morocco was based on phenotyping data of 288 lines. The selected GS model for Algeria, which was based on one-year data from Algiers, was Ridge Regression BLUP with up to 3,354 markers per cross, showing an average intra-environment, intra-cross predictive ability of 0.18 (Annicchiarico et al., 2020). The selected GS model for Morocco was based on line mean yield across the environments of Marchouch and managed severe drought in Lodi, which shared very high drought stress, very similar site mean yield, and fairly similar line adaptive responses (Annicchiarico et al., 2020). This model was Bayesian Lasso with up to 8,773 markers per cross, showing an average intra-environment, intra-cross predictive ability of 0.69 (Annicchiarico et al., 2020).

The region-specific GS was applied to a common set of 720 lines, which encompassed 120 lines per cross that were bulk-derived from natural and mass selection under field conditions in Northern Italy and 120 lines per cross that were bulk-derived from mass selection under managed drought (**Figure 2**). For each region, we selected the two top-yielding lines per cross according to genomically-predicted breeding values (**Figure 3**).

Only for GS, we envisaged a fourth putative target region named hereafter “Stressful Italy”, which could roughly represent the drought-prone areas of Southern Italy. Predictions for this putative region were performed for each cross by averaging the genome-enabled predictions issued by the models for Italy and for Morocco, selecting two lines per cross (**Figure 3**).

### Evaluation of the selected material

The evaluated material included (**Figure 3**): (a) three evolutionary populations, one per region (Central Italy, Algeria, Morocco); (b) nine phenotypically-selected lines, three per region (of which one per cross), that were regionally top-yielding in earlier selection trials; (c) 24 genomically-selected lines, six per region (of which two per cross) for each of the regions of Central Italy, Algeria, Morocco, and the putative stressful Italy region, including lines that were top-yielding according to relevant region-specific GS models. The evaluated entries actually amounted to 33 (hereafter globally referred to as genotypes) instead of 36, because two lines were genomically selected for both Morocco and Stressful Italy, and one was genomically selected for both Central Italy and Stressful Italy. All of these lines and the three parent cultivars (Attika, Isard, and Kaspa) were evaluated for grain yield and a few additional traits in autumn-sown, pure stand trials performed in Perugia, Algiers and Marchouch in the seasons 2021–22 and 2022-23, and in a managed drought stress experiment carried out in Lodi in 2021-22.

In addition, the 10 genotypes (one EP; three lines from PS; six lines from GS) that were specifically selected for Algeria, and the 10 genotypes that were specifically selected for Morocco, were evaluated in their relevant target regions for grain yield in intercropping with barley. These trials were performed in 2021–22 and 2022–23 in Marchouch, and in 2021–22 in Algiers (where a second trial in 2022–23 failed due to technical problems). The barley cultivars, chosen on a regional basis, were Ferdaouss in Morocco and Fouara in Algeria.

The trials in Algiers and Marchouch were designed as a RCB with three replications for both pure stand and intercropping. These growing conditions were spatially arranged as main plots of a split-plot design with respect to the 10 region-specific selections, to better highlight GEI effects across pure stand and mixed stand environments. Each plot had 1.5 m^2^ size. Pure stand crops included 120 seeds in 4 rows (plant density: 80 seeds/m^2^). Mixed stand plots had pea and barley seeds mixed on the row, with seed densities of 40 seeds/m^2^ for pea and 150 seeds/m^2^ for barley. The pre-sowing fertilization provided all crops with 30 kg/ha each of N, P_2_O_5_, and K_2_O. The trials in Perugia were designed and managed likewise but included only the pure stand condition. Rainfall and absolute minimum temperature for these trials are given in **Table 1**.

The managed drought stress experiment in Lodi was performed in a large phenotyping platform with a rain-out shelter and micro-irrigators that comprised two possible soil types: sandy-loam, and silty-clay. The experiment, sown in autumn and designed as a RCB with two replicates within each soil type environment, adopted a plot size of 0.6 × 0.8 m with 40 plants (plant density: 83.33 plants/m^2^) and the same pre-sowing fertilization as the other experiments. The overall amount of water was greater than in previous experiments under managed drought conditions (**Table 1**), as it aimed to represent a moderately drought-prone environment.

We recorded the dry seed yield of pea and barley on a plot basis, after estimating seed moisture on a random sample of 100 seeds per plot for pea and 250 seeds per plot for barley after oven drying at 60°C for four days. We recorded the following traits in pure stand trials: (a) onset of flowering (as the number of days from March 1 to when 50% of plants in the plot had at least one open flower), in all environments; (b) straw production at harvest, and (c) plant height at the beginning of flowering as average canopy height, both observed in five environments (Algiers and Marchouch in both years, and the managed stress environment); (d) single seed dry weight based on 100 random oven-dried seeds per plot, observed in five environments (Algiers and Perugia in both years, and the managed stress environment). Tolerance to lodging, winter low temperatures and pests or diseases was not recorded, owing to the limited extent of these constraints.

### Statistical analysis of experiment data

We compared SSD, bulk selection under drought (as the predominant stress), and bulk selection under low winter temperatures (as the predominant stress) for ability to provide top-yielding lines in the following five contrasting environments or locations: (a) managed severe drought stress in 2015; (b) Marchouch in 2015-16; (c) Algiers in 2015-16; (d) Perugia in 2013-14; and (e) Lodi across the seasons 2013–14 and 2014-15. Given the different number of evaluated SSD-derived and bulk-derived lines, we did not compare a fixed number of top-yielding lines per plant material (which would favor the material with more evaluated lines, i.e., that from SSD) but a fixed proportion of them, namely, the 20% top-yielding lines per material, generating results that were averaged across the three crosses. Operationally, we first identified in each test environment or location the 20% top-yielding lines for each plant material and cross – which resulted in 12 lines per cross for SSD, and six or three lines per cross for bulk selections (in case of 30 or 15 evaluated lines, respectively). Then, we compared the three plant materials for mean yield of their top-yielding set in: (a) four single environments, namely, the managed stress, Marchouch, Algiers, and Perugia, by an analysis of variance (ANOVA) including the fixed factors material, cross, and line within material and cross, and the random factor block (ANOVA model 1 in **Supplementary Table 1**); (b) Lodi across two cropping years, by an ANOVA including, in addition, the factor year (ANOVA model 2 in **Supplementary Table 1**).

A preliminary ANOVA performed on data of the managed drought stress experiment in 2021–22 indicated the absence of GEI for genotype yield across environments represented by the two soil types (ANOVA model 3 in **Supplementary Table 1**). Therefore, this experiment was analyzed as a RCB with four blocks (two from each environment) in following ANOVAs.

We performed an ANOVA on pea yield data of the three locations (Algiers, Marchouch, Perugia) that included, besides the random factor block within location and year, the following four fixed factors: (a) germplasm type, with 11 variants, of which nine were defined by the factorial combinations of three plant materials (EP, PS-derived line, GS-derived line) by three target regions (Algeria, Morocco, Central Italy), one included GS-derived lines for Stressful Italy, and one included the parent germplasm; (b) genotype within germplasm type; (c) location; (d) year, considered as fixed because 2021–22 and 2022–23 represented for all sites a low-yielding and a high-yielding cropping year, respectively (ANOVA model 4 in **Supplementary Table 1**).

Other ANOVAs on pea yield data encompassed: (a) an ANOVA comparing plant materials in each year, including plant material (EP, PS-derived line, and GS-derived line), genotype within material, and location as fixed factors, and block within location as random factor (ANOVA model 5 in **Supplementary Table 1**); (b) ANOVAs comparing plant materials within each target region (ANOVA model 6 in **Supplementary Table 1**) or the managed stress environment (ANOVA model 7 in **Supplementary Table 1**), which included plant material, genotype within material, and year (limitedly to locations) as fixed factors, and block (within year for locations) as random factor; (c) ANOVAs comparing genotypes within each target region (with factors genotype, year, and block within year; ANOVA model 8 in **Supplementary Table 1**) or under managed drought (with factors genotype and block; ANOVA model 9 in **Supplementary Table 1**).

We investigated the adaptation of the individual genotypes across seven pure stand stand environments represented by the combinations of three locations (Algiers, Marchouch, and Perugia) by two test years plus the managed drought stress environment. We performed a combined ANOVA on pea grain yield including the fixed factors genotype and environment and the random factor block within environment (ANOVA model 3 in **Supplementary Table 1**). GEI was partitioned by Additive Main effects and Multiplicative Interaction (AMMI) analysis, testing the significance of GEI principal component (PC) axes by the *F_R_* test (Piepho, 1995). We reported a biplot of genotype and environment scores in the space of the first two GEI PC axes, and investigated by correlation analysis the relationships of environment PC scores with climate variables and that of genotype PC scores with morphophysiological traits averaged over environments. Genotype adaptive responses were expressed as AMMI-modelled nominal yields (which exclude the site main effect, irrelevant for entry ranking) as a function of the environment score on the first GEI PC (Gauch et al., 2008). For sake of clarity, we produced one graph with mean values of the 11 germplasm types, and another graph including the EPs, the parent lines, and the lines from PS or GS that were among the three top-yielding lines in at least one environment. Considering the relatively high number of environments that is needed for a reliable assessment of yield stability (Piepho, 1998), we assessed genotype yield stability by the Environmental Variance across seven environments. We combined mean yield and yield stability into an index of yield reliability by estimating the lowest genotype yield expected in 80% of cases (Eskridge, 1990). This reliability threshold represented a compromise between values around 75% proposed for modern agriculture and values around 90% proposed for subsistence agriculture (Eskridge, 1990).

The yield gains provided by each selection strategy were assessed separately for each target region, comparing the EP and the top-yielding lines from GS and PS that were selected for the region with the regionally top-yielding parent cultivar. For the same material, we also assessed the genetic gains for yield reliability based on yield values across seven environments.

One ANOVA aimed to compare germplasm types for onset of flowering, straw production, plant height, and individual seed weight across environments. It included germplasm type, genotype within germplasm type, and environment as fixed factors, and block within environment as random factor (ANOVA model 10 in **Supplementary Table 1**).

Other ANOVAs were limited to the 10 regional selections evaluated in the conditions of pure stand and mixed stand in Algiers and Marchouch. For Algiers, an ANOVA including the factors cropping condition, genotype, and block assessed the occurrence of GEI across conditions for pea yield according to the split-plot lay-out (ANOVA model 11 in **Supplementary Table 1**), whereas a second ANOVA compared the genotypes for pea yield in the pure stand environment and for pea yield, barley yield and pea proportion in the mixed stand environment (ANOVA model 9 in **Supplementary Table 1**). For Marchouch, where the experiment in intercropping was repeated for two years, the ANOVAs included also the fixed factor year (according to ANOVA models 12 and 8 in **Supplementary Table 1** respectively for the ANOVA with and without the cropping condition factor).

The AMMI analysis was performed using the software CropStat (IRRI, 2009), computing genotype nominal yields by subtracting the environment main effect from the modelled yields in each environment that were outputted by the software for the AMMI model including one GEI PC axis as described in Annicchiarico (2002). The remaining statistical analyses were carried out by SAS software Version 9.4 (SAS Institute, 2016), using the PROC GLM for all ANOVAs.

## Results

### Comparison of SSD-derived vs. bulk-derived lines, and phenotypic and genomic selections

The yield comparison based on the 20% locally top-yielding lines between SSD-derived lines and bulk-derived lines generated either under managed severe drought or under low winter temperatures in autumn-sown environments of Northern Italy was performed in five environments, among which Marchouch and Lodi were the most contrasting in water availability and absolute minimum temperature (**Table 1**). The advantage of bulk-derived lines over SSD-derived ones increased in environments characterized by a high level of the predominant stress that had been imposed on the bulked plants. In particular, the top-performing lines bulk-selected under drought conditions out-yielded the top-performing SSD-derived lines by 14.7% (*P* < 0.05) in the managed severe drought environment (the driest one), by 10.5% (*P* < 0.10) in Marchouch, and only by 5.6% (not significant) in Algiers (**Table 2**). Likewise, the top-performing lines bulk-selected at low winter temperatures out-yielded the top-performing SSD-derived lines by 19.1% (*P* < 0.05) in Lodi (the coldest site), and by 14.2% (*P* < 0.05) in Perugia (**Table 2**). The importance of evolutionary adaptation to specific conditions was confirmed by the misadaptation of (a) the top-performing lines bulk-selected under low winter temperatures in the evaluations performed in Marchouch or under managed drought, and (b) the top-performing lines bulk-selected under managed drought when evaluated under field conditions in Lodi. In these cases, these bulk-derived materials exhibited a yield penalty of at least 11% (*P* < 0.05) compared to SSD-derived material (**Table 2**).

**Table 2:** Mean grain yield in five environments of the 20% top-yielding inbred lines issued from bulk selection (BS) under either of two major stresses or single-seed descent (SSD). Values averaged across results for three connected crosses, based on data of 288 to 306 evaluated lines.

Environment/Origin of inbred lines	Yield (t/ha)^a^
Managed severe drought	
BS, severe drought^b^	0.602 a
SSD	0.525 b
BS, low winter temperatures^c^	0.231 c
Marchouch (severe drought)	
BS, severe drought^b^	0.693 a
SSD	0.627 a
BS, low winter temperatures^c^	0.489 b
Algiers (moderate drought)	
BS, severe drought^b^	2.284 a
SSD	2.163 a
BS, low winter temperatures^c^	2.085 a
Perugia (moderate drought and winter cold)	
BS, severe drought^b^	3.589 b
SSD	3.801 b
BS, low winter temperatures^c^	4.340 a
Lodi (winter cold)	
BS, severe drought^b^	5.909 c
SSD	6.651 b
BS, low winter temperatures^c^	7.925 a

a Means within environment followed by different letter differ at *P* < 0.05 according to Duncan’s test.

b Bulk selection performed in Lodi under managed severe drought over three years.

c Bulk selection performed in Lodi under autumn-sown field conditions over four years.

Marchouch and Algiers showed a wide variation in rainfall amount in the years used for PS (**Table 1**). In Algeria and Central Italy, two of the three lines finally selected by PS derived from bulk breeding under the relevant predominant stress, implying four-fold greater probability of PS for bulk-derived lines compared to those derived from SSD (**Supplementary Table 2**). In Morocco, however, bulk-derived and SSD-derived lines exhibited the same probability of final PS (**Supplementary Table 2**).

Most of the genomically-selected lines derived from bulk breeding under the relevant predominant stress, namely, four lines out of six in Algeria, five out of six in Morocco, and all six lines in Central Italy (**Supplementary Table 2**). Of the six lines selected genomically for Stressful Italy by averaging the results of GS models for Italy and Morocco, three originated from bulk breeding under drought and three from bulk breeding under low winter temperatures (**Supplementary Table 2**).

### Yielding ability, yield stability, adaptation, and morphophysiological traits of selected material

On average, the Moroccan site was drier and lower yielding than Algiers, which, in turn, was warmer and somewhat drier and lower yielding than Perugia (**Tables 1**, **3**). The first evaluation year (2021-22) was significantly less productive than the second (2022-23) for all locations (**Table 3**), mainly because of lower rainfall during the crop cycle for Marchouch and Perugia (**Table 1**) and less favorable rainfall distribution for Algiers. The mean yield in the managed moderate drought environment was intermediate but closer to that of sites in the favorable year (**Table 3**).

**Table 3:** Mean grain yield (t/ha) in seven test environments for the whole set of genomically-selected or phenotypically-selected lines, evolutionary populations, and parent cultivars.

Cropping year	Algiers (Algeria)	Marchouch (Morocco)	Perugia (Italy)	Managed drought (Lodi)
2021-22	1.61 d	0.45 e	1.80 d	3.99 b
2022-23	4.49 a	2.49 c	4.93 a	−

Means followed by different letter differ at *P* < 0.05 according to Duncan’s test.

In the ANOVA for pea yield in the three locations, the 11 germplasm types defined by parent germplasm and region-specific EPs, lines from PS, and lines from GS did not differ for mean yield but showed significant (*P* < 0.05) first- and second-order interactions with location and year (**Supplementary Table 3**). Genotypes within germplasm type differed in mean yield but showed first- and second-order interaction with location and year (*P* < 0.001; **Supplementary Table 3**), indicating the large extent of GEI in this dataset. The results in **Table 4** provided insight on the germplasm type × year interaction in relation to the plant materials selected for Algeria, Morocco or Central Italy: on average, EP material out-yielded GS- and PS-derived materials in the climatically unfavorable year (*P* < 0.05), while the three plant materials showed no differences in the favorable year.

**Table 4:** Mean grain yield (t/ha) across three locations of coastal Algeria, inland Morocco and Central Italy in the cropping years 2021–22 and 2022–23 of genomically-selected (GS) or phenotypically-selected (PS) inbred lines and evolutionary populations (EP) targeted to these regions.

Material	No. of entries	2021-22	2022-23
Lines from GS	18	1.298 b	3.951 a
Lines from PS	6	1.285 b	3.994 a
EP	3	1.488 a	3.809 a
LSD (*P* < 0.05)		0.129	0.228

Column means followed by different letter differ at *P* < 0.05 according to Duncan’s test.

According to results in **Table 5**, germplasm type × location interactions were affected only to some extent by specific adaptation of the germplasm to its target region, which emerged mainly for materials selected for Central Italy. The comparison of the three plant materials (EP, PS-derived lines, and GS-derived lines) for mean yield at each location revealed only a few significant differences (*P* < 0.05) that did not concern, anyway, the materials selected and evaluated in the same region (**Table 5**). However, important indications emerged from comparisons limited to the EP and the top-yielding lines from GS or PS selected specifically for each region (**Table 5**). In Algeria, the top-yielding line from GS out-yielded both the top-yielding line from PS and the EP (*P* < 0.05). In Morocco, the top-yielding line from GS out-yielded the EP (*P* < 0.05) while showing a non-significant advantage over the top-yielding line from PS. In Central Italy, the top-yielding lines from GS or PS performed comparably and out-yielded the EP (*P* < 0.05). Central Italy was the only region where the top-yielding parent cultivar was significantly out-yielded (*P* < 0.05) by top-performing lines from GS or PS (**Table 5**). In the managed stress environment, the top-yielding germplasm type was the EP selected in Morocco (which was top-ranking also in Algiers and second-ranking in Perugia), whereas the top-yielding genotype was a line genomically selected for both Morocco and Stressful Italy (which was top-yielding also in Marchouch) (**Table 5**).

**Table 5:** Mean grain yield (t/ha) of plant materials and yield of the top-yielding line in three locations and under managed drought, for genomically-selected (GS) or phenotypically-selected (PS) inbred lines and evolutionary populations (EP) targeted to regions of Algeria, Morocco or Italy and their parent cultivars (results for locations averaged across two test years).

Target region	Material	No. of entries	Algiers (coastal Algeria)		Marchouch (inland Morocco)		Perugia (Central Italy)		Managed drought	
			Mean	Top-yielding	Mean	Top-yielding	Mean	Top-yielding	Mean	Top-yielding
Coastal Algeria	Lines from GS	6	2.984	4.101	1.401	1.717	3.365	3.742	4.119	4.432
Coastal Algeria	Lines from PS	3	3.174	3.316	1.402	1.617	3.324	3.623	4.422	4.589
Coastal Algeria	EP	1	2.802	−	1.461	−	3.443	−	4.115	−
Inland Morocco	Lines from GS	6	3.252	3.682	1.450	1.922	3.206	3.741	4.356	4.885
Inland Morocco	Lines from PS	3	3.143	3.418	1.346	1.700	3.301	3.828	4.132	4.649
Inland Morocco	EP	1	3.378	−	1.400	−	3.692	−	4.649	−
Central Italy	Lines from GS	6	2.838	3.439	1.623	1.800	3.448	4.396	3.508	3.957
Central Italy	Lines from PS	3	2.668	3.474	1.480	1.699	3.780	4.480	3.302	4.157
Central Italy	EP	1	2.857	−	1.304	−	3.499	−	3.502	−
Stressful Italy	Lines from GS	6	3.046	3.817	1.642	1.922	3.448	3.741	4.212	4.885
−	Parent cultivars	3	3.290	3.726	1.411	1.611	2.960	3.068	3.842	4.901
LSD (*P* < 0.05)^a^			0.463^a^	0.659^b^	0.291^a^	0.439^b^	0.376^a^	0.535^b^	0.463^a^	0.664^b^

a For the comparison of groups of material.

b For the comparison of individual genotypes.

**Figure 4:**
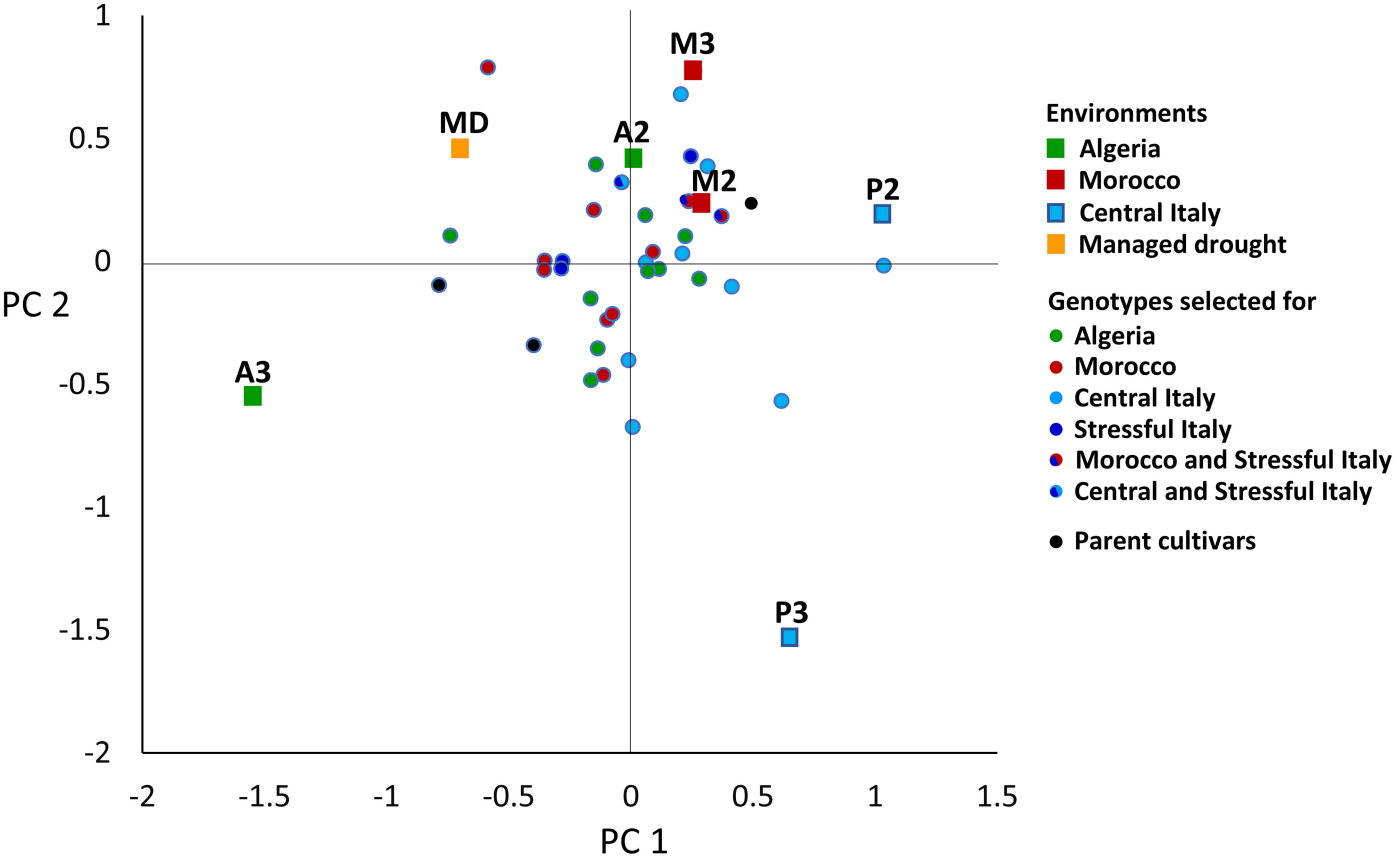
Biplot of 36 genotypes and 7 environments in the space of the first (PC 1) and second (PC 2) genotype × environment interaction principal component axes. A2 and A3, Algiers (coastal Algeria); cropping years 2021–22 and 2022-23; M2 and M3, Marchouch (inland Morocco); years 2021–22 and 2022-23; P2 and P3, Perugia (Central Italy); years 2021–22 and 2022-23; MD, managed drought.

**Figure 5:**
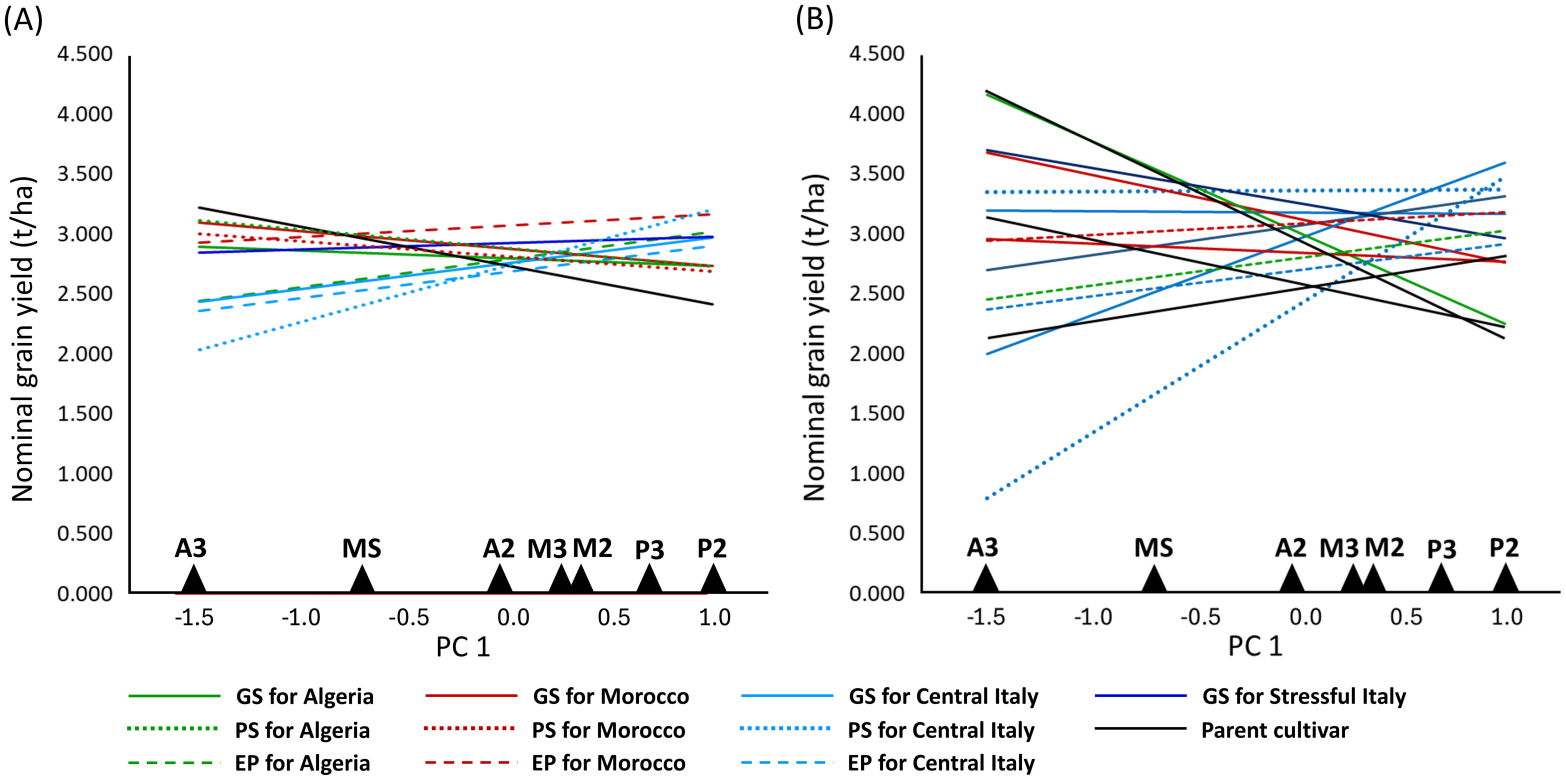
Nominal grain yield as a function of the first genotype × environment interaction principal component score (PC 1) of seven environments for 11 germplasm types relative to genomically-selected (GS) or phenotypically-selected (PS) inbred lines and evolutionary populations (EP) targeted to coastal Algeria, inland Morocco or Central Italy, lines from GS for Stressful Italy, and three parent cultivars. **(A)** reports EPs and the mean values of the parent cultivars and the lines from GS and PS for each target region; **(B)** reports EPs, parent cultivars, and the lines from PS or GS that were among the three top-yielding lines in at least one environment.

In the AMMI analysis of pea yield in seven environments, three GEI PC axes were significant at *P* < 0.01, of which PC 1 explained 39%, PC 2 26%, and PC 3 15% of the overall GEI variation (**Supplementary Table 4**). PC 1 was an indicator of winter cold stress and PC 2 an indicator of drought stress of the environments, based on the negative correlations of the environment PC 1 score with the absolute minimum temperature (*r* = −0.86, *P* < 0.05) and the environment PC 2 score with December to May rainfall (*r* = −0.69, *P* < 0.10). The environments belonging to the same location were not very close in the space of these PC axes (**Figure 4**), reflecting the substantial genotype × year interaction that emerged in a previous ANOVA (**Supplementary Table 3**). The managed moderate drought environment was roughly intermediate between the Algerian and Moroccan environments for genotype adaptive responses (**Figure 4**). The genotype ordination along PC 1 was correlated with later onset of flowering (*r* = 0.58, *P* < 0.01) and, to a lesser extent, greater straw production (*r* = 0.39, *P* < 0.05) and taller plant stature (*r* = 0.30, *P* < 0.10) (in the presence of correlation ≥ 0.72 between these morphophysiological traits). A trend towards a positive GEI of genotypes grown in their target environments emerged more clearly for material targeted to Central Italy in the biplot of **Figure 4** (as indicated by a closer distance of relevant genotype-environment pairs in the graph) and according to the nominal yield responses of germplasm types or genotypes as a function of the environment PC 1 score (**Figure 5**). A trend towards low GEI was shown by: (a) the lines selected genomically for Stressful Italy (**Figures 4**, **5A**); (b) the three EPs, which generally showed lower specific adaptation to their target region than the lines issued from PS or GS (**Figure 5A**). The nominal yield responses of a subset of locally best-yielding lines, the individual parent cultivars and the EPs (**Figure 5B**) highlighted the presence of some lines from GS or PS and one parent cultivar (Attika) with locally higher yield but generally more restricted adaptability compared to the EPs.

On average, the plant materials selected for Algeria, Morocco or Central Italy exhibited comparable mean yield but different yield stability, which was highest (as indicated by lowest Environmental Variance) for the EPs and lowest for the parent cultivars (**Table 6**). As a result, the plant materials could be ranked in the order EPs > lines from GS or PS > parent cultivars in terms of yield reliability (**Table 6**). However, the genotype variation within plant materials was substantial, and some lines issued from GS or PS displayed yield stability and yield reliability comparable to that of EP material (**Table 6**). On average, the lines selected genomically for Stressful Italy displayed yield reliability comparable with that of the EPs, owing to top-ranking mean yield and to yield stability intermediate between the EPs and other lines (**Table 6**).

**Table 6:** Mean and range values of grain yield, yield stability expressed as Environmental Variance, and yield reliability expressed as lowest yield expected in 80% of cases, estimated for genomically-selected (GS) or phenotypically-selected (PS) inbred lines and evolutionary populations targeted to regions of Algeria, Morocco or Italy and their parent cultivars, based on data from seven environments represented by six location-year combinations for Algiers, Marchouch and Perugia plus a managed drought stress environment.

		Yield (t/ha)		Environmental Variance (t/ha)^2^		Yield reliability (t/ha)	
Material	No. of lines	Mean	Range	Mean	Range	Mean	Range
Lines from GS^a^	18	2.815	2.454 - 3.170	3.024	1.898 - 4.250	1.361	1.149 - 1.679
Lines from PS^a^	9	2.814	2.395 - 3.352	3.133	1.631 - 4.665	1.347	1.157 - 1.538
Evolutionary populations^a^	3	2.852	2.689 - 3.077	2.582	2.311 - 2.932	1.504	1.412 - 1.638
Lines from GS for Stressful Italy	6	2.926	2.590 - 3.250	2.820	2.220 - 3.440	1.521	1.273 - 1.692
Parent cultivars	3	2.738	2.530 - 3.033	3.419	2.041 - 4.606	1.205	1.054 - 1.330

^a^Pooling the material targeted to coastal Algeria, inland Morocco and Central Italy.

Yield gains of the EP and the top-yielding lines from GS and PS bred for each region relative to the regionally top-yielding parent cultivar are given in **Table 7**. For both Algeria and Morocco, only GS was able to produce germplasm with a distinct yield gain (> 10%). For Central Italy, GS, PS applied to bulk-derived material, and the EP were all able to provide a distinct gain (≥ 14%), but GS and PS provided a much greater gain (> 43%). **Table 7** also reports the yield reliability values of the regionally top-yielding genotypes based on data of the seven environments. Due to their high yield stability, the EPs exhibited yield reliability values roughly comparable with those of the best lines issued from GS or PS for the same region. These genotypes showed a distinct gain in yield reliability (> 14%) compared to the top-yielding cultivars.

**Table 7:** Grain yield across two cropping years in their target region, yield reliability across seven environments, and yield gains (%) over the regionally top-yielding parent cultivar, for the evolutionary population and the regionally top-yielding inbred lines issued from genomic selection (GS) and single-seed descent (SSD)-derived or bulk-derived phenotypic selection (PS) targeted to regions of Algeria, Morocco or Italy.

Material	Yield (t/ha)		Yield reliability (t/ha)^a^	
	Value^b^	Gain	Value	Gain
Selections for coastal Algeria				
Line from GS	4.101 a	10.1	1.387	12.7
SSD-derived line from PS	3.316	-11.0	1.366	11.0
Bulk-derived line from PS	3.232	-13.3	1.289	4.7
Evolutionary population	2.802	-24.8	1.461	18.7
Top-yielding parent (Attika)	3.726 a	−	1.231	−
Average of parent cultivars	3.290	−	−	−
Selections for inland Morocco				
Line from GS	1.922 a	19.3	1.551	16.6
SSD-derived line from PS	1.700 a	5.5	1.402	5.4
Bulk-derived line from PS	1.023	-36.5	1.157	-13.0
Evolutionary population	1.400	-13.1	1.638	23.2
Top-yielding parent (Kaspa)	1.611 a	−	1.330	−
Average of parent cultivars	1.411	−	−	−
Selections for Central Italy				
Line from GS	4.396 a	43.3	1.419	15.3
SSD-derived line from PS	3.082	0.5	1.391	13.0
Bulk-derived line from PS	4.480 a	46.0	1.538	24.9
Evolutionary population	3.499	14.0	1.412	14.7
Top-yielding parent (Attika)	3.068	−	1.231	−
Average of parent cultivars	2.960	−	−	−

a As lowest yield expected in 80% of cases, estimated across six location-year combinations for Algiers, Marchouch and Perugia plus a managed drought stress environment.

b Genotype values within each regional selection followed by letter ‘a’ do not differ from the top-ranking value at *P* < 0.05 according to Duncan’s test.

The morphophysiological traits of the 11 germplasm types averaged across evaluation environments are reported in **Table 8**. In general, the regional selections ranked in the order Central Italy > Algeria > Morocco for values of onset of flowering, plant height, and straw production. On average, parent lines and lines issued from GS for Stressful Italy tended to display intermediate values of these traits. EP and PS-derived material consistently tended towards greater straw production than GS-derived lines bred for the same region. The EP material was taller for Central Italy and Morocco and earlier flowering for Algeria compared to inbred lines for the same region, while showing inconsistent results for individual seed weight (relatively high in North African countries and low in Central Italy). The material from PS showed taller stature, later flowering, and definitely larger seed than GS-derived material only for Italy.

**Table 8:** Agronomic traits of genomically-selected (GS) or phenotypically-selected (PS) inbred lines and evolutionary populations (EP) targeted to regions of Algeria, Morocco or Italy and their parent cultivars.

Target region	Material	Onset of flowering (dd from March 1)^a^	Straw production (t/ha)^b^	Plant height (cm)^b^	Individual seed weight (g)^b^
Coastal Algeria	Lines from GS	35.8	5.654	63.5	0.171
Coastal Algeria	Lines from PS	34.3	5.996	65.6	0.176
Coastal Algeria	EP	32.6	6.192	60.3	0.183 a
Inland Morocco	Lines from GS	30.8 b	4.927 b	55.3 b	0.181 a
Inland Morocco	Lines from PS	31.5 b	5.500	54.5 b	0.171
Inland Morocco	EP	31.6 b	5.549	60.9	0.183 a
Central Italy	Lines from GS	38.6	5.883	63.6	0.153 b
Central Italy	Lines from PS	41.9 a	6.807 a	68.0	0.171
Central Italy	EP	40.8	7.064 a	71.9 a	0.155 b
Stressful Italy	Lines from GS	33.9	5.607	57.6	0.166
−	Parent cultivars	34.9	5.573	58.9	0.173
LSD (*P* < 0.05)		0.9	0.520	2.9	0.005

Column means followed by the letter ‘a’ and ‘b’ do not differ from the top-ranking and bottom-ranking mean, respectively, according to Duncan’s test.

a Evaluated in seven environments.

b Evaluated in five environments.

### Adaptation to intercropping of selected material

Pea was out-competed by barley in both regions, as indicated by an average pea content on total grain yield of 17.1% in Algeria and 33.9% in Morocco. The ANOVAs for pea yield of 10 region-specific genotypes grown under conditions of pure stand or mixed stand with barley revealed GEI across conditions for both Algeria (*P* < 0.01) and Morocco (*P* < 0.05) (**Supplementary Table 5**). Non-significant correlations for pea genotype yield across pure stand and mixed stand (*r* = 0.33 for Algeria; *r* = 0.10 for Morocco) confirmed the inconsistency of the genotype yield responses across these growing conditions. Pea genotype variation was found for pea yield and pea proportion in mixed stand and for pea yield in pure stand in both regions (*P* < 0.05; **Supplementary Table 6**). Genotype variation for yield of the associated cereal was found only in Algeria, where, however, the lack of inverse correlation between pea and cereal yield of the genotypes (*r* = 0.10) indicated that higher pea yield was not detrimental to cereal yield of the mixture. Actually, a similar conclusion was supported for Morocco by the absence of genotype variation for yield of the associated cereal in the presence of variation for pea yield in mixed stand.

For both regions, EP material showed greater adaptability to intercropping than lines from GS or PS according to (a) its pea yield advantage in mixed stand (*P* < 0.05) in the absence of yield advantage in pure stand, and (b) its greater proportion of pea in mixed stand (*P* < 0.05) (**Table 9**). Even the best-performing line in mixed stand could, at most, approach the pea yield and pea proportion of the EP in mixed stand, a result in contrast with the yield advantage of the best-performing line over the EP in pure stand (**Table 9**).

**Table 9:** Pea grain yield in pure stand, and pea yield, barley yield and pea grain proportion in mixed stand, of genomically-selected (GS) or phenotypically-selected (PS) inbred lines and evolutionary populations (EP) targeted to coastal Algeria or inland Morocco in their respective target regions (data for one cropping year in Algeria and two cropping years in Morocco).

Material	No. of lines	Pea yield in pure stand (t/ha)	Pea yield in mixed stand (t/ha)	Barley yield in mixed stand (t/ha)	Pea grain proportion in mixed stand
Selections for coastal Algeria					
Lines from GS	6	1.389 a	0.477 b	3.051 a	0.135 b
Lines from PS	3	1.740 a	0.664 b	2.674 a	0.198 b
EP	1	1.735 a	1.236 a	2.917 a	0.298 a
Top-yielding line in pure stand		2.162	0.430	3.917	0.099
Top-yielding line in mixed stand		1.734	0.950	2.699	0.260
Selections for inland Morocco					
Lines from GS	6	1.450 a	0.908 b	1.843 a	0.330 b
Lines from PS	3	1.346 a	1.018 a	2.039 a	0.333 b
EP	1	1.400 a	1.153 a	1.605 a	0.418 a
Top-yielding line in pure stand		1.922	0.959	1.696	0.361
Top-yielding line in mixed stand		1.314	1.132	1.970	0.365

Mean values of lines from GS, lines from PS and EP followed by different letter differ at *P* < 0.05 according to Duncan’s test.

## Discussion

### Comparison of SSD-derived vs. bulk-derived lines

Our study encourages the exploitation of evolutionary adaptation and mass selection of bulked segregating material under growing conditions similar to those of the target environments of the breeding program, to increase the agronomic value of the lines subjected to subsequent PS or GS. This was supported, in particular, by the comparison of locally top-yielding germplasm sets across five climatically contrasting environments, and was confirmed by the origin of the lines that were regionally selected by PS or GS. Bulk breeding shaped substantially the adaptive responses of the genotypes after three or four generations of natural and/or mass selection. The greatest advantage of bulk-derived lines over SSD-derived ones was achieved in environments characterized by a similar type and magnitude of the predominant stress that had acted on the bulked plants. Some degree of mismatching occurred in all cases between environment(s) used for bulk breeding and the target environments (Northern Italy, to breed for Central Italy; managed severe drought, to breed for Algeria or Morocco), reflecting the practical limitation of using one specific site or growth condition for bulk breeding. A closer matching between these environments may have increased the probability for relevant bulk-derived material to be phenotypically selected.

The advantage of bulk-derived over SSD-derived lines confirms earlier results for pea that are mainly relative to Northern Italy (Annicchiarico et al., 2023). As anticipated, earlier comparisons of bulk-derived vs. SSD-derived lines provided inconsistent results for grain yield of legume crops (Haddad and Muehlbauer, 1981; Kumar et al., 1992; Meena and Kumar, 2012). We could hypothesize four possible reasons contributing to the current advantage of bulk-derived material. One is the performance of within-bulk yield-based mass selection (in one late generation for Italy and in every generation for North African regions) instead of relying solely on natural selection, a procedure that proved beneficial also in Meena and Kumar (2012). Secondly, the presence of one predominant environmental stress acting on bulked plants, a condition that favored the bulk selection of chickpea for resistance to Ascochyta blight (*Fusarium* spp.) or to low winter temperatures (Singh et al., 1994). Thirdly, the attention that we paid to ensuring a balanced intra-specific plant competition in early generations. Finally, our choice of parent cultivars with similar, semi-dwarf plant stature. Such a choice prevented the presence of large within-cross variation for plant stature and the prevalence in the evolving population of taller genotypes with greater intra-specific competition for light combined with lower grain yielding ability due to lower investment in reproductive development (Hamblin and Rowell, 1975; Khalifa and Qualset, 1975).

### Genotype × environment interaction patterns

The wide year-to-year climatic variation of the evaluation sites favored the occurrence of large genotype × year and genotype × location × year interactions for pea yield, which also emerged in earlier studies for Italy (Annicchiarico and Iannucci, 2008; Pecetti et al., 2019). Large GEI for pea yield was also reported across Mediterranean regions (Iglesias-García et al., 2017). The large year-to-year variation for rainfall amount that occurred in experiments used for PS complicated the region-specific selection and limited the exploitation of region-specific adaptation for each target region. The occurrence of region-specific adaptation emerged mainly for material bred for Central Italy. AMMI analysis results suggested that the extent of winter cold stress was a key determinant of specific-adaptation patterns, with later flowering acting as a frost escape mechanism (Maqbool et al., 2010) conferring specific adaptation to the colder and more moisture-favorable environments of Central Italy. Accordingly, the EP evolved in this region had distinctly later onset of flowering than the other EPs. Greater straw production and taller plant stature showed a loose relationship with specific adaptation to Central Italy in the AMMI analysis, and featured the EP evolved in this region, possibly as an indirect effect of (a) their correlation with later flowering or (b) their relationship with greater competitive ability against weeds (McDonald, 2003) in the organically-managed environments adopted for PS trials and EP development in this region.

Our comparison of selection strategies was mainly based on the yielding ability of the genotypes in their target region, but also included information on the yield stability and reliability of the genotypes (which necessarily relied on all evaluation environments) because of (a) the large genotype × year interaction and its expected increase due to increasing within-region year-to-year climatic variation and (b) the limited number of evaluation environments representing each target region.

The competitive disadvantage of pea intercropped with cereals and the substantial GEI for pea yield across pure stand and mixed stand conditions, which emerged consistently across the experiments of Algiers and Marchouch, are frequent in pea-cereal mixtures (Annicchiarico et al., 2019a, Annicchiarico et al., 2021; Haug et al., 2023) and are in agreement with the general trend towards a large GEI of legume-based mixtures in which the legume is definitely out-competed (Annicchiarico et al., 2019a). The display by more competitive pea genotypes of increased pea yield without a decrease in cereal yield emphasizes the importance of legume competitive ability for both balance and total yield of the mixture. Such a yield response is common when the legume component is at competitive disadvantage and can be attributed to more nitrogen made available to the cereal and other complementarity effects (Annicchiarico et al., 2019a).

### Comparison of genomic vs. phenotypic selection of inbred lines

Earlier studies encouraged the GS for pea grain yield on the basis of predictive ability values and the possible comparison of GS vs. PS based on predicted yield gains (Annicchiarico et al., 2019b, Annicchiarico et al., 2020; Crosta et al., 2022; Saludares et al., 2024; Crosta et al., 2025). This study provides an unprecedented comparison of GS vs. PS in terms of inbred lines actually developed from a common genetic base, with results covering three distinct target regions. The GS- and PS-derived lines did not differ in mean yield in their target regions or in mean yield, yield stability and yield reliability across environments. However, the comparison based on top-yielding material in its target region revealed the superiority of GS for Algeria and a tendency in this direction for Morocco, whereas GS and PS were comparable for Central Italy. GS was the only selection strategy that allowed for a distinct genetic gain in all target regions. The good performance of GS for Algeria (particularly when focusing on its top-yielding line) was remarkable considering that the average intra-cross predictive ability of GS for this region was low (only 0.18) compared with the average intra-environment, intra-cross predictive ability for the other regions (≥ 0.48; Annicchiarico et al., 2019b, Annicchiarico et al., 2020). However, the assessment of GS-derived material for Algeria benefited from a good consistency for climatic characteristics between environment(s) used for GS definition and the evaluation environments of the selected materials (**Table 1**). A lower consistency occurred especially for Central Italy, where two environments used for GS definition out of three were from Northern Italy, and one evaluation environment exhibited particularly low rainfall (**Table 1**). In all regions, GS may have suffered a slight penalty compared to PS due to the fact that the 30 top-yielding lines in the last mass-selection stage of bulk breeding were used for PS (and GS model definition) while applying GS to lower-ranking lines issued from mass selection. On the other hand, PS was limited by performing its large-scale evaluation stage in only one environment for Algeria and in two environments of Northern Italy out of three when targeting Central Italy (**Figure 3**), and by the modest number of lines that were promoted beyond the first PS stage. Indeed, a reason contributing to the comparable response of top-yielding PS and GS lines in Central Italy might be the two-fold greater number of lines promoted to three-environment PS in this region (**Figure 3**).

The only morphophysiological difference between GS and PS that emerged consistently was the higher straw production of PS material, which may be relevant for crop-livestock systems due to the moderate forage quality of the pea straw (Carrouée et al., 2003). One could speculate that the lower straw yield of GS material may be due to a high implicit emphasis on higher harvest index in the GS models aimed at improving grain yield.

The low GEI and high yield stability and reliability shown by the lines selected genomically for the putative Stressful Italy region supports the use of GS based on models for contrasting environments as a means to breed material with wide climate adaptation and high and stable grain yield. This finding is of special interest in the context of the increasing year-to-year climatic variation associated with climate change.

### Comparison of evolutionary population vs. inbred line material

The comparison based on mean values of the plant materials indicated that the EPs did not differ from GS- or PS-derived lines in their target regions or across regions, while showing (a) greater yield stability and reliability, (b) better response in climatically-unfavorable years, and (c) broader adaptability to non-target environments such as intercropping or, particularly for the Moroccan EP, other non-target environments such as coastal Algeria, Central Italy, and the managed stress environment. However, each EP showed a yield penalty when compared with the top-yielding inbred line selected specifically for its target region and the target growing condition of pure stand. Therefore, the elite inbred line material was able to maximize the crop yielding ability in its target environments, while possessing narrower adaptability to non-target conditions than the EP material.

In a prior pea study, an EP that evolved in Northern Italy and top-yielding bulk-derived lines issued from PS had shown a similar yielding ability in Northern Italy (Annicchiarico et al., 2023). In general, the present results are in agreement with the greater yield stability and similar or somewhat lower mean yield of EPs compared to top-performing parent lines or other inbred line material that was reported for cool-season cereals (e.g., Döring et al., 2015; Bocci et al., 2020; Goldringer et al., 2020; Merrick et al., 2020; Baresel et al., 2022). The broader adaptability and yield stability of EPs compared to inbred lines was expected as a result of intra-specific diversity. The particularly large adaptability of the EP selected in Morocco may have been enhanced by the exceptional year-to-year rainfall variation (from 51 to 410 mm) that occurred during its evolution in this region, which may have favored the coexistence of genotypes with contrasting adaptability in the population.

Intra-specific diversity may also have contributed to the greater adaptation to intercropping of EP material compared to top-yielding lines. Greater within-species diversity is expected to favor the species coexistence and balance in plant communities (Vellend, 2006), although its positive effect on pea intercropped with cereals was reportedly modest in Darras et al. (2015).

The EPs from Morocco and Central Italy exhibited taller stature, which could enhance the competitive ability of pea against cereals in intercropping or against weeds in low-input systems. The top-performing straw production showed by EPs compared to regionally-selected inbred lines reinforce the interest of this variety type for crop-livestock systems. The trend towards taller stature and greater straw production displayed by the EPs agrees with outcomes for cool-season cereals (Goldringer et al., 2001; Knapp et al., 2020) and could be expected as a result of intra-specific competition for light during their evolution. The inconsistent results for individual seed weight in the comparison between EP and inbred line material are difficult to explain, for a trait on which the effect of natural selection is controversial (Horneburg and Becker, 2008; Goldringer et al., 2001).

### Implications of the results for breeding strategies

A biotechnology-based approach such as GS, and agroecology-based approaches such as bulk breeding and EP selection, may serve different purposes and offer different opportunities depending on the size, infrastructure, resource allocation and target environments of the breeding programs. Our results encourage the development of region-specific EPs especially for private or public breeding programs with modest size and resources that target low-input systems and/or areas where crop yields tend to be lower and more erratic due to harsh and highly variable climatic conditions. EPs are less expensive to select than inbred line varieties and could be valuable for increasing the grain yield stability and reliability and for widening the within-region adaptation to new cropping systems, as indicated by the current results for intercropping. The high production of pea straw could be a valuable additional asset of EP material for climatically-unfavorable areas and/or subsistence agriculture, where livestock production is severely limited by the availability of forage resources (FAO, 2010). The EP variety type could be seen as a sort of “modern landrace”, with potential for further adaptation to farm-specific pedoclimatic and crop management conditions. The growing scientific awareness of the value of genetically heterogeneous material justifies the development of policy frameworks that increase their marketing opportunities within formal seed systems (Joshi et al., 2024). However, EPs can already be registered and marketed for organic farming in the EU, while informal systems offer important opportunities for their marketing in developing countries (Sperling and Almekinders, 2023), including those of the Maghreb (Sadiki et al., 2004).

**Figure 6:**
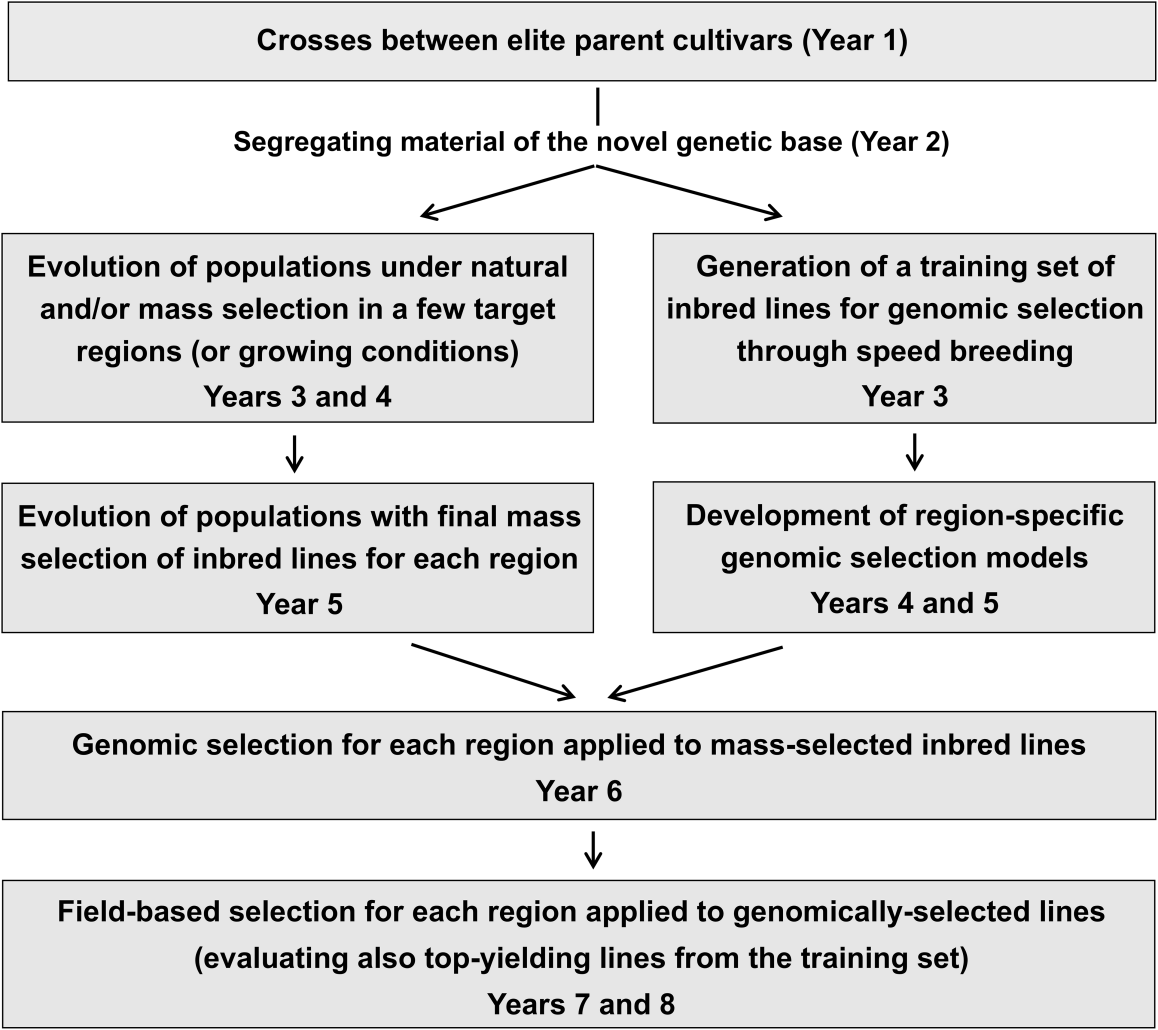
Example of integration of bulk breeding, speed breeding, and genomic selection for selection of higher-yielding pea inbred lines.

Our findings may also serve as inspiration for large-scale breeding programs with adequate infrastructure to implement cost-efficient solutions for inbred line selection. They indicated (a) the higher genetic gain per selection cycle in specific target environments of bulk-derived over SSD-derived lines, obtained at lower cost for bulk breeding (especially when relying mainly on natural selection), and (b) the higher genetic gain per unit time of GS over PS, when a GS model is available. On the other hand, the implementation of bulk breeding in large and well-funded selection programs is limited by the slow generation of inbred material compared to SSD, which may rely on off-season generations possibly based on speed breeding (Mobini and Warkentin, 2016). Likewise, the implementation of GS is constrained by the time needed to build the GS model based on phenotyping data from a training population of lines. Both constraints could largely be overcome by a selection strategy that is exemplified in **Figure 6**. We suggest taking advantage of speed breeding to promptly produce a representative training set of the target genetic base to be subjected to multi-year phenotyping for GS model construction, while exploiting the advantages of bulk breeding to simultaneously generate inbred lines on which the GS model will be applied. An interesting opportunity offered by GS is the combination of different models to select material with a broader climatic adaptation, as demonstrated by the current selections targeting the Stressful Italy region.

When based exclusively on PS, breeding programs could include solutions that combine the advantages of bulk-based selection in a few, early segregating generations followed by speed breeding or off-season generation of its mass-selected lines.

Genomic selection and evolutionary breeding are not incompatible and may be integrated in large breeding programs by producing EPs of bulk-derived material evolved under specific conditions to be used as a valuable dynamic management of genetic resources for future selection.

Some of the current selections exhibited high yielding ability, yield stability and adaptability compared with the commercial cultivars that acted as parent germplasm (**Table 7**). A few top-yielding lines are undergoing additional testing before being proposed for registration in the target countries. The EP selected for Central Italy is going to be notified as Organic Heterogeneous Material. The EPs for Algeria and Morocco are going to be distributed to farmers in the respective countries via informal seed systems.

## Data Availability

The datasets presented in this study can be found in online repositories. The names of the repository/repositories and accession number(s) can be found below: https://figshare.com/, 28254347.
