# Supplementary material for: Comparison of phenotypic selection of inbred lines, genomic selection of inbred lines, and evolutionary populations for field pea breeding in three Mediterranean regions
Source: Front Plant Sci. 2025 Jun 17;16:1565087. doi: 10.3389/fpls.2025.1565087 (PMC12209206; doi:10.3389/fpls.2025.1565087)
Supplement: Supplementary file 1 [file Table1.docx]

**Supplementary Table 1**. **Analysis of variance (ANOVA) models used for analyses. R, response variable; M, plant material; C, cross; T, germplasm type; G, genotype or line; E, environment; L, location; Y, year; Cr, cropping condition (pure or mixed stand); B, randomized complete block; e, (pooled) experiment error.**

| ANOVA model | Model effects |  |
| --- | --- | --- |
| 1 | R = M + C + G(M C) + B + M×C + C×B + e |  |
| 2 | R = M + C + G(M C) + Y + B(Y) + M×C + M×Y + C×Y + G(M C)×Y + C×B(Y) + e |  |
| 3 | R = G + E + B(E) + G×E + e |  |
| 4 | R = T + G(T) + L + Y + B(L Y) + T×L + T×Y + T×L×Y + G(T)×L + G(T)×Y + G(T)×L×Y + L×Y + e^a^ |  |
| 5 | R = M + G(M) + L + B(L) + M×L + G(M)×L + e^a^ |  |
| 6 | R = M + G(M) + Y + B(Y) + M×Y + G(M)×Y + e^a^ |  |
| 7 | R = M + G(M) + B + e^a^ |  |
| 8 | R = G + Y + B(Y) + G×Y + e |  |
| 9 | R = G + B + e |  |
| 10 | R = T + G(T) + E + B(E) + T×E + G(T)×E + e^a^ |  |
| 11 | R = G + Cr + B + G×Cr + Cr×B^b^ + e |  |
| 12 | R = G + Cr + Y + B(Y) + G×Cr + G×Y + Cr×Y + G×Cr×Y + Cr×B(Y)^b^ + e |  |

^a^ The interactions with B of T and G(T), or those of M and G(M), were pooled together in the pooled experiment error, since the genotypes of the different germplasm types or plant materials were randomized together within each block.

^b^ Separated and used as an error term for Cr according to the split-plot lay-out.
