# Supplementary material for: Comparison of phenotypic selection of inbred lines, genomic selection of inbred lines, and evolutionary populations for field pea breeding in three Mediterranean regions
Source: Front Plant Sci. 2025 Jun 17;16:1565087. doi: 10.3389/fpls.2025.1565087 (PMC12209206; doi:10.3389/fpls.2025.1565087)
Supplement: Supplementary file 2 [file Table2.docx]

**Supplementary Table 2. Origin of lines selected phenotypically or genomically for four target regions: number and percentage of lines derived from bulk selection (BS) under either of two major stresses or from single-seed descent (SSD).**

|  | Phenotypic selection^a^ | |  | Genomic selection^b^ | |
| --- | --- | --- | --- | --- | --- |
| Target region | Number | Percentage |  | Number | Percentage |
| *Coastal Algeria* |  |  |  |  |  |
| BS, severe drought^c^ | 2 | 6.67 |  | 4 | 1.11 |
| SSD | 1 | 1.67 |  | − | − |
| BS, low winter temperatures^d^ | 0 | 0.00 |  | 2 | 0.55 |
| *Inland Morocco* |  |  |  |  |  |
| BS, severe drought^c^ | 1 | 3.33 |  | 5 | 1.39 |
| SSD | 2 | 3.33 |  | − | − |
| BS, low winter temperatures^d^ | 0 | 0.00 |  | 1 | 0.28 |
| *Central Italy* |  |  |  |  |  |
| BS, severe drought^c^ | 0 | 0.00 |  | 0 | 0.00 |
| SSD | 1 | 1.67 |  | − | − |
| BS, low winter temperatures^d^ | 2 | 6.67 |  | 6 | 1.67 |
| *Stressful Italy* |  |  |  |  |  |
| BS, severe drought^c^ | − | − |  | 3 | 0.83 |
| BS, low winter temperatures^d^ | − | − |  | 3 | 0.83 |

^a^ Algeria and Morocco: selection performed on 30 lines from BS under severe drought, 15 lines from BS under low winter temperatures, and 60 lines from SSD; Italy: selection performed on 15 lines from BS under severe drought, 30 lines from BS under low winter temperatures, and 60 lines from SSD.

^b^ Selection performed on 360 lines from BS under low winter temperatures and 360 lines from BS under low winter temperatures.

^c^ Bulk selection performed in Lodi under managed severe drought over three years.

^d^ Bulk selection performed in Lodi under autumn-sown field conditions over four years.
