# Supplementary material for: Comparison of phenotypic selection of inbred lines, genomic selection of inbred lines, and evolutionary populations for field pea breeding in three Mediterranean regions
Source: Front Plant Sci. 2025 Jun 17;16:1565087. doi: 10.3389/fpls.2025.1565087 (PMC12209206; doi:10.3389/fpls.2025.1565087)
Supplement: Supplementary file 3 [file Table3.docx]

**Supplementary Table 3**. **Analysis of variance for grain yield of 36 pea genotypes belonging to 11 germplasm types grown in three locations (Algiers, Marchouch, Perugia) for two years.**

| Source of variation | Degrees of freedom | Sum of squares | Mean square | *F* test^a^ |  |
| --- | --- | --- | --- | --- | --- |
| Germplasm type (T)^b^ | 10 | 2.20 | 0.22 | NS |  |
| Genotype (G) within T | 28 | 36.17 | 1.29 | *** |  |
| Location (L) | 2 | 402.47 | 201.23 | *** |  |
| Year (Y) | 1 | 979.88 | 979.88 | *** |  |
| L × Y | 2 | 29.09 | 14.54 | ** |  |
| Block within L × Y | 12 | 22.99 | 1.92 |  |  |
| T × L | 20 | 17.36 | 0.87 | *** |  |
| T × Y | 10 | 4.745 | 0.47 | * |  |
| T × L × Y | 20 | 8.47 | 0.42 | * |  |
| G × L within T | 56 | 48.71 | 0.87 | *** |  |
| G × Y within T | 28 | 23.97 | 0.86 | *** |  |
| G × L × Y within T | 56 | 39.89 | 0.71 | *** |  |

^a^ NS, not significant at *P* < 0.05; *, **, ***, significant at *P* < 0.05, *P* < 0.01 and *P* < 0.001, respectively.

^a^ Represented by combinations of plant material and target region as defined in Table 4.
