# Supplementary material for: Comparison of phenotypic selection of inbred lines, genomic selection of inbred lines, and evolutionary populations for field pea breeding in three Mediterranean regions
Source: Front Plant Sci. 2025 Jun 17;16:1565087. doi: 10.3389/fpls.2025.1565087 (PMC12209206; doi:10.3389/fpls.2025.1565087)
Supplement: Supplementary file 4 [file Table4.docx]

**Supplementary Table 4**. **Additive Main Effects and Multiplicative Interaction analysis of grain yield for 36 pea genotypes grown in seven environments.**

| Source of variation | Degrees of freedom | Sum of squares | Mean square | *F* test^a^ |  |
| --- | --- | --- | --- | --- | --- |
| Genotype (G) | 35 | 46.54 | 1.33 | *** |  |
| Environment (E) | 6 | 1858.10 | 309.68 | *** |  |
| Block within E | 15 | 37.79 | 2.52 |  |  |
| G × E interaction | 210 | 164.19 | 0.78 | *** |  |
| G × E interaction PC 1 | 40 | 63.71 | 1.59 | *** |  |
| G × E interaction PC 2 | 38 | 42.74 | 1.12 | *** |  |
| G × E interaction PC 3 | 36 | 24.23 | 0.67 | *** |  |
| Residual G × E interaction | 96 | 33.51 | 0.35 | NS |  |
| Pooled experiment error | 525 | 132.41 | 0.25 |  |  |

^a^ NS, not significant at *P* < 0.05; ***, significant at *P* < 0.001.
