# Supplementary material for: Comparison of phenotypic selection of inbred lines, genomic selection of inbred lines, and evolutionary populations for field pea breeding in three Mediterranean regions
Source: Front Plant Sci. 2025 Jun 17;16:1565087. doi: 10.3389/fpls.2025.1565087 (PMC12209206; doi:10.3389/fpls.2025.1565087)
Supplement: Supplementary file 5 [file Table5.docx]

**Supplementary Table 5**. **Analysis of variance *F* test results for pea grain yield in the cropping conditions of pure stand and mixed stand with barley, for two different sets of 10 pea genotypes grown in coastal Algeria for one cropping year and inland Morocco for two years.**

| Source of variation | Algeria | Morocco |  |
| --- | --- | --- | --- |
| Genotype | ** | ** |  |
| Condition | * | ** |  |
| Year | − | ** |  |
| Genotype × Condition | * | ** |  |
| Genotype × Year | − | * |  |
| Genotype × Condition × Year | − | ** |  |

*, significant at *P* < 0.05; **, significant at *P* < 0.01.
