# Supplementary material for: Comparison of phenotypic selection of inbred lines, genomic selection of inbred lines, and evolutionary populations for field pea breeding in three Mediterranean regions
Source: Front Plant Sci. 2025 Jun 17;16:1565087. doi: 10.3389/fpls.2025.1565087 (PMC12209206; doi:10.3389/fpls.2025.1565087)
Supplement: Supplementary file 6 [file Table6.docx]

**Supplementary Table 6**. **Analysis of variance *F* test results for pea grain yield in pure stand and pea grain yield, cereal grain yield and pea proportion in pea-barley mixed stand, for two different sets of 10 pea genotypes grown in coastal Algeria for one cropping year and inland Morocco for two years.**

| Trait | Algeria | Morocco |  |
| --- | --- | --- | --- |
| Pea grain yield in pure stand | ** | ** |  |
| Pea grain yield in mixed stand | ** | * |  |
| Cereal grain yield in mixed stand | * | NS |  |
| Pea proportion in mixed stand | * | * |  |

NS, not significant at *P* < 0.05; *, significant at *P* < 0.05; **, significant at *P* < 0.01.
